# Co-Designing an Antiracist Dental Health System: Protocol for an Aboriginal and Torres Strait Islander–Led Mixed Methods Study

**DOI:** 10.2196/69012

**Published:** 2025-05-15

**Authors:** Brianna Poirier, Joanne Hedges, Dandara Haag, Yin Paradies, Tamara Mackean, João Bastos, Catherine Leane, Gustavo Soares, Sneha Sethi, Jessica Manuela, Pedro Santiago, Kelli Owen, Natalie Bauer, Jodie Milne, Ashleigh Smith, Kelly Smith, Priscilla Larkins, Madison Cachagee, Vaibhav Garg, Latisha Sykora, Nicolas Reid, Michael Larkin, Jayde Fuller, Lisa Jamieson

**Affiliations:** 1 Australian Research Centre for Population Oral Health, Adelaide Dental School University of Adelaide Adelaide Australia; 2 Alfred Deakin Institute for Citizenship and Globalisation Deakin University Burwood Australia; 3 Flinders Health and Medical Research Institute Flinders University Adelaide Australia; 4 Faculty of Health Sciences Simon Fraser University Burnaby, BC Canada; 5 Aboriginal Health Division Women's and Children's Health Network North Adelaide Australia; 6 Indigenous Dental Association of Australia Melbourne Australia; 7 Faculty of Health and Medical Science University of Adelaide Adelaide Australia; 8 Yadu Health Aboriginal Corporation Ceduna Australia; 9 Moorundi Aboriginal Community Controlled Health Service Murray Bridge Australia; 10 Umoona Tjutagku Health Service Coober Pedy Australia; 11 Royal Flying Doctor Service of Australia SA&NT Adelaide Australia; 12 Aboriginal and Torres Strait Islander Health Strategy Unit Australian Health Practitioner Regulation Agency Melbourne Australia

**Keywords:** racism, anti-racism, dentistry, Aboriginal and Torres Strait Islander, dental workforce, dental curriculum, oral health promotion, Aboriginal Community Controlled Health Organizations, Aboriginal Health Workers/Practitioners

## Abstract

**Background:**

Racism arrived in Australia with colonization and its intentionally oppressive policies and actions toward Aboriginal and Torres Strait Islander Peoples. To a large extent, colonial and biomedical agendas are maintained by Australia’s health system that underlies much of the racialized health inequities in the country. Dentistry significantly lags behind medicine and other health care areas in the uptake of antiracism, with the dental accreditation body only acknowledging racism as a determinant of oral health in 2022.

**Objective:**

This project will comprehensively co-design strategies for an antiracist dental health system in Australia through the following objectives: (1) development of an antiracist curriculum for dental students; (2) workforce strategies that support the attraction, retention, and well-being of the Aboriginal and Torres Strait Islander dental workforce; and (3) oral health promotion training for Aboriginal Health Workers/Practitioners.

**Methods:**

This project is grounded in decolonizing and Indigenous methodologies, which guide our ways of working at the knowledge interface. Co-design Yarning sessions will inform the development and implementation associated with each of the objectives through tabulation and narrative synthesis of sessions. Objectives will be evaluated with both quantitative and qualitative measures and analyzed accordingly with inverse probability of treatment weighting, content analysis, or reflexive thematic analysis.

**Results:**

The study received ethical review approval in February 2024 and received funding in June 2024. The co-design phase for each objective will run from July 2024 to February 2025. The dental curriculum will be developed in 2025 and delivered to the 2026 student cohort. Evaluation data will be collected from the comparator student cohort in 2025 and the implementation cohort in 2026. Data collection for the development of workforce strategies will be collected from October 2024 to July 2025; the framework will be developed from August to December 2025 and disseminated in 2026. Oral health promotion training will be developed from August to February 2024, implemented from March to June 2025, and qualitative evaluation data will be collected between July and September 2025.

**Conclusions:**

The proposed research will develop an antiracism curriculum for dental students, a tailored Aboriginal and Torres Strait Islander dental workforce framework, and an oral health promotion training program for Aboriginal Health Workers/Practitioners. The processes and final outcomes of this research will be scalable and able to be tailored to different contexts. Together, these strategies will build oral health knowledge at the Community level, in turn supporting Aboriginal and Torres Strait Islander self-determination of oral health.

**International Registered Report Identifier (IRRID):**

PRR1-10.2196/69012

## Introduction

It is widely accepted that social determinants of health [1,2], and for some, commercial determinants of health [3], directly impact one’s oral health outcomes. There remains a disconnect between the role structural factors play in shaping oral health among clinicians, public servants, and researchers [4]. Beyond debating the use of race as an indicator for certain dental procedures [5] or to assess racial disparities in oral health outcomes [6], limited action has been taken to address the role structural racism plays in creating oral health inequities [7,8]. Both cultural and structural racism emerge within dentistry, impacting oral health outcomes in several ways. At a cultural level, racism is perpetuated through interpersonal racism and implicit bias, whereby clinicians discriminate against patients on the basis of race and ethnicity [9]. At a structural level, racism manifests as institutional practices, policies, and ideologies that inherently afford advantages to some racial groups over others [10,11]. Racism is, in fact, a dental public health threat due to its intentional deprivation of access to health equity and sovereignty (the rights and abilities for Communities to make their own decisions about all aspects of their lives), further entrenching cycles of disease burden within racially oppressed communities [9].

Racism arrived on the shores of Australia in 1788 with colonization and the intentional dehumanization of Aboriginal and Torres Strait Islander Peoples by settler colonists [12,13]. Colonization violently established whiteness as the societal and human ideal in Australia, and indeed in other settler colonial nations, based on the rights of “white possession” and individual sovereignty [14]. Aboriginal and Torres Strait Islander Peoples had better oral health outcomes than non-Indigenous people up until the 1970s, which demonstrates that the oral health inequities are grounded in persistent racialized ideologies of colonial care [15]. The institutionalization of care within Australia via state welfare maintains “White ignorance” [16] and colonial ideologies within the reality of ongoing and systemic racial violence [17]. While medicine and other areas of health care have been slow in the uptake of an antiracist agenda into their conduct of practice, dentistry significantly lags behind [7,9]. Internationally, only a few antiracism initiatives have been undertaken within dentistry, including 1 training program for dental students [18] and 1 regulatory framework [19]; there have been no Australian-based initiatives. The accreditation process for all dental practitioners, as established by the Australian Dental Council, failed to name racism as a determinant of oral health until 2022 [20]. This exemplifies the racialized foundations of the Australian dental health system and provides rationale for why dental decay remains the most prevalent chronic condition experienced by Aboriginal and Torres Strait Islander Peoples across all age groups [21]. Racism embedded in Australia’s infrastructure is not restricted to the dental health system but manifests across mutually reinforcing structures, from child removal and incarceration [22] to missing and murdered women and children [23]. Widespread and ongoing stereotyping across all aspects of Australian society perpetuates racialized discourses about Aboriginal and Torres Strait Islander Peoples and leads to embedded discrimination in systems experienced when accessing care, which negatively affects access to health care and culminates in negative health outcomes [24]. There remains a relational and moral obligation to counter racism in its various forms, particularly in the dental context where moral ignorance has fed the normalization of racism as the status quo since dentistry’s inception [25].

Structural racism in Australia’s dental health system creates differentials in professional leadership, workforce representation, advocacy, and access to services. For example, while Aboriginal and Torres Strait Islander Peoples represent 3.8% of the Australian population, as of 2020, only 0.4% of all practicing dentists were Aboriginal and/or Torres Strait Islander. Across Australia’s 9 dental schools and peak national dental bodies, none of these institutions are led by an Aboriginal and/or Torres Strait Islander person. Such a degree of underrepresentation is one of the largest among all health professions [26], positioning dentistry significantly behind the targets of the National Aboriginal and Torres Strait Islander Health Workforce Strategic Framework and Implementation Plan [27]. These imbalances created and perpetuated by structural and cultural forms of racism also normalize expressions of interpersonal racism in dental clinical settings. Research indicates that patients’ race affects providers’ clinical decisions [28], which affects quality of care. Due to power imbalances between students and educators or patients and clinicians, oral health practitioners in Australia are rarely accountable for discriminating against Aboriginal and Torres Strait Islander patients, reflecting the inability of the dental health system to provide culturally safe care. This intensifies oral disease inequities, as evidenced by lower dental attendance for Aboriginal and Torres Strait Islander patients [29]. Subsequently, oral health practitioners’ assumptions that racially minoritized patients do not give importance to oral health are reinforced by the racial ideologies that initially influenced oral health inequities. The impact of oral diseases spans across the life course, affecting outcomes such as identity, chronic diseases, lost economic productivity, social and emotional well-being, as well as difficulty finding a job [2]. The Australian dental system’s inability to properly address the oral health needs of Aboriginal and Torres Strait Islander Communities demonstrates how oral health inequities are rooted in racialized systems that continue to perpetuate a cycle of trauma and intergenerational disadvantage for Aboriginal and Torres Strait Islander Peoples.

It is critical to apply collaborative solutions that tackle racism within Australia’s dental health system and promote the delivery of culturally safe and quality dental care for Aboriginal and Torres Strait Islander Communities [7]. Grounded in decolonial methodologies, we will comprehensively co-design strategies that mitigate the impacts of racism across different contexts within Australia’s dental health system.

### Study Aims

This project aims to implement evidence through co-designed strategies that apply best practices for dental health system changes and foster the provision of antiracist dental care in Australia through the following objectives (Textbox 1).

**Co-designed objectives.**

- Develop and implement an antiracist curriculum for dental students that can be robustly evaluated. The benefits include an evidence-based model and pedagogical framework for a curriculum that will provide the necessary foundation for antiracist practice across Australia’s dental health system.
- Generate a framework of strategies to be implemented across a number of professional settings (ie, dental organizations, dental schools, and dental clinics) to support the attraction, retention, and well-being of the Aboriginal and Torres Strait Islander dental workforce. The benefits include fostering support and growth of the Aboriginal and Torres Strait Islander dental workforce in Australia.
- Create and evaluate an oral health promotion training module for Aboriginal Health Workers/Practitioners (AHW/P). The benefits include supporting Aboriginal and Torres Strait Islander well-being by sharing oral health promotion information in a manner that equips AHW/P with knowledge to have everyday yarns about oral health with Community members.

## Methods

### Positionality Statement

Our appreciation of the importance of positionality and relationality has largely been influenced by decolonizing frameworks and Indigenous methodologies [30,31]. As a collective coming to this work, we intentionally position ourselves within these processes to acknowledge our connections, relationships, and responsibilities [32-34]. As a team comprised of Aboriginal and Torres Strait Islander researchers, dental professionals, Community leaders and non-Indigenous researchers, we are grateful for the opportunity we share to work alongside Aboriginal and Torres Strait Islander Communities. We are led by a proud Yamatji woman and a Project Governance Committee (PGC) consisting of 25 Aboriginal and Torres Strait Islander leaders from across so-called Australia. The supporting team consists of Indigenous and non-Indigenous members from Turtle Island, Australia, Brazil, India, and Aotearoa. The strength of this team is in our diverse perspectives and experiences related to racism, our shared values, and our commitments to antiracist health care, equity, social justice, and self-determination. The intentional development of this team facilitates Aboriginal and Torres Strait Islander guidance in decision-making, leadership in data collection and analysis, and ensures robust interpretation of findings. We remain grateful for our relational foundations of this work and committed to (un)learning throughout our individual and collective life-long journeys of antiracism.

### Methodology

#### Decolonizing Methodologies

**Figure 1:**
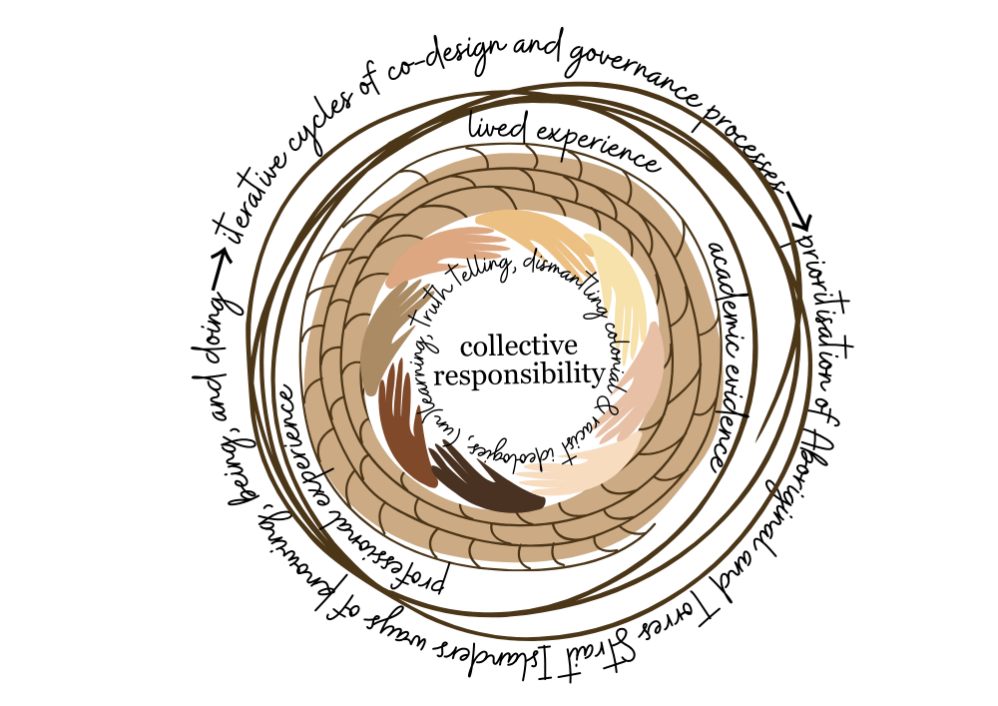
Ways of working conceptual model that weaves together lived experiences, academic evidence, and professional experiences throughout iterative processes that center around shared values.

This study will be informed by decolonizing methodologies, and therefore, grounded in an understanding of settler colonialism in Australia and resistance to colonial marginalization from dominant culture through assertions of individual and collective sovereignty [35-37]. Whereby, dominant culture is understood as aligning with colonial and biomedical values and health systems. In decentring colonial values, this project will follow processes that center Aboriginal and Torres Strait Islander identity, cultural and collective action, as well as self-determination [38,39]. Decolonizing methodologies counteract colonial research processes that are embedded in a history of appropriation, exploitation, misrepresentation, and unethical practices [31,39-42]. The generation, validation, and dissemination of knowledge through colonial research practices enable control of dominant ideologies that subordinate Indigenous knowledge systems [40,43]. The failure of colonial research practices to adequately address Aboriginal and Torres Strait Islander experiences of inequitable health is fundamentally related to the power held by those who determine the value of knowledge and research processes [38]. Therefore, decolonizing methodologies will be used in this project to explicitly acknowledge the impacts of colonization and settler colonialism, resist dominant deficit discourses of Aboriginal and Torres Strait Islander ill health [44], and advance Aboriginal and Torres Strait Islander health sovereignty. In the context of dentistry, this requires our team and our project challenge statements and beliefs rooted in colonial and racial ideologies, demand truth-telling, and collectively commit to (un)learning. As Aboriginal and Torres Strait Islander research leadership and governance is critical in ensuring respectful, reciprocal, and culturally safe practices, this project will be governed by a PGC. In undertaking this work from a decolonial standpoint, we acknowledge our continued reliance on colonial processes for findings to have translatable impacts, particularly with regard to advocacy for health system reform. Therefore, we will equally value and weave together 3 types of evidence: lived experiences, professional experience, and academic evidence. We recognize that research will never be entirely “decolonized,” as research itself is rooted in colonial foundations; however, we contend that there remains an ethical compulsion to create and maintain spaces that drive Aboriginal and Torres Strait Islander–led programs (Figure 1).

#### Indigenous Research Methodologies

Employing Indigenous research methodologies and methods supports decolonial values and processes by shifting paradigms and power from colonial academic institutions to Aboriginal and Torres Strait Islander ways of knowing, being, and doing [45]. Indigenous research methodologies necessitate embedded relationality from project inception through to sharing of findings [30,37]; the notion of relationality is represented by the hands sharing our collective responsibilities in Figure 1. The iterative process of co-design Yarning with external stakeholders and internal team members underpins the entire project. Yarning sessions among the research team, throughout the co-design phase, and beyond will dynamically move through the Yarning Process developed by Bessarab, from Social Yarns to Research, Work, and Topic Yarns, as well as Collaborative and Therapeutic Yarns, and back again [46]. This commitment to continuous and relational Yarning [47,48] is critical when exploring (anti)racism due to the need for ongoing individual and collective reflexivity [49] and the creation of a spaces that foster agency for individuals to share information and opinions at their discretion [37]. Our approach to this research is guided by our relationships, prioritizes Indigenous research methods, is accountable to and informed by Aboriginal and Torres Strait Islander Communities, and is led by our Aboriginal and Torres Strait Islander PGC [50].

The PGC is comprised of leaders from partnering organizations, with whom we have established relationships, to ensure that all aspects of the project are conducted in a culturally safe manner that prioritizes self-determination, demonstrates respect, and privileges Aboriginal and Torres Strait Islander ways of knowing, being, and doing [37,45]. Membership includes Aboriginal Community Controlled Health Organizations (ACCHOs) representatives, Elders, Aboriginal and Torres Strait Islander dentists, current Aboriginal and Torres Strait Islander dental students, Community leaders, representatives from regulatory and organizational dental bodies, and members of the research team. Aboriginal and Torres Strait Islander members of the PGC are honored not only in their roles as academics, health practitioners, or Community leaders but also for their roles as mothers, fathers, aunties, uncles, and grandparents with lived experiences and vested interest in the oral health of their families and Communities. The PGC will provide governance and input into curriculum development, training development for Aboriginal Health Workers/Practitioners (AHW/P), data interpretation, and dissemination of findings. PGC meetings will be held quarterly throughout the duration of the project, with feedback sought outside of meetings on an ongoing basis. The PGC will be comprised of more than 75% Aboriginal and Torres Strait Islander leaders and will ensure that project processes and outcomes remain relevant and in alignment with Community values, needs, and experiences (Multimedia Appendix 1).

#### Knowledge Interface

Working at the knowledge interface [51] within colonial research spaces using decolonial and Indigenous research methodologies allows different knowledge systems to be brought together with the aim of generating contextual evidence and novel insights. This requires reciprocal and trusting partnerships that center, rather than marginalize, Aboriginal and Torres Strait Islander ways of knowing, being, and doing [45]. We will prioritize a collaborative approach to knowledge creation through iterative co-design processes across all project objectives whereby lived experiences, professional experiences, and academic evidence are valued equally, which is visually represented by the hands supporting our collective responsibility in Figure 1. To navigate the epistemological tensions that exist when working in decolonial ways at the knowledge interface, as a team we will continuously engage in individual and collective reflexivity through weekly team meetings, independent journaling, and supporting (un)learning processes. Reflexivity requires each member of the team, individually and as a collective, to examine, interrogate, and reflect on our beliefs, feelings, and actions and how they are influencing decision-making. This process of engaging in decolonial knowing has previously been termed “dialoguing with the tensions,” and is a critical step in moving from individual immersion in resources and knowledges to collective and transformative discussions and action [52]. Positioning our project at the knowledge interface is a significant strength of our approach, as this enables the prioritization of decolonial ideologies and Aboriginal and Torres Strait Islander ways of knowing, being, and doing within colonial academic institutions and biomedical health systems.

### Study Design

**Figure 2:**
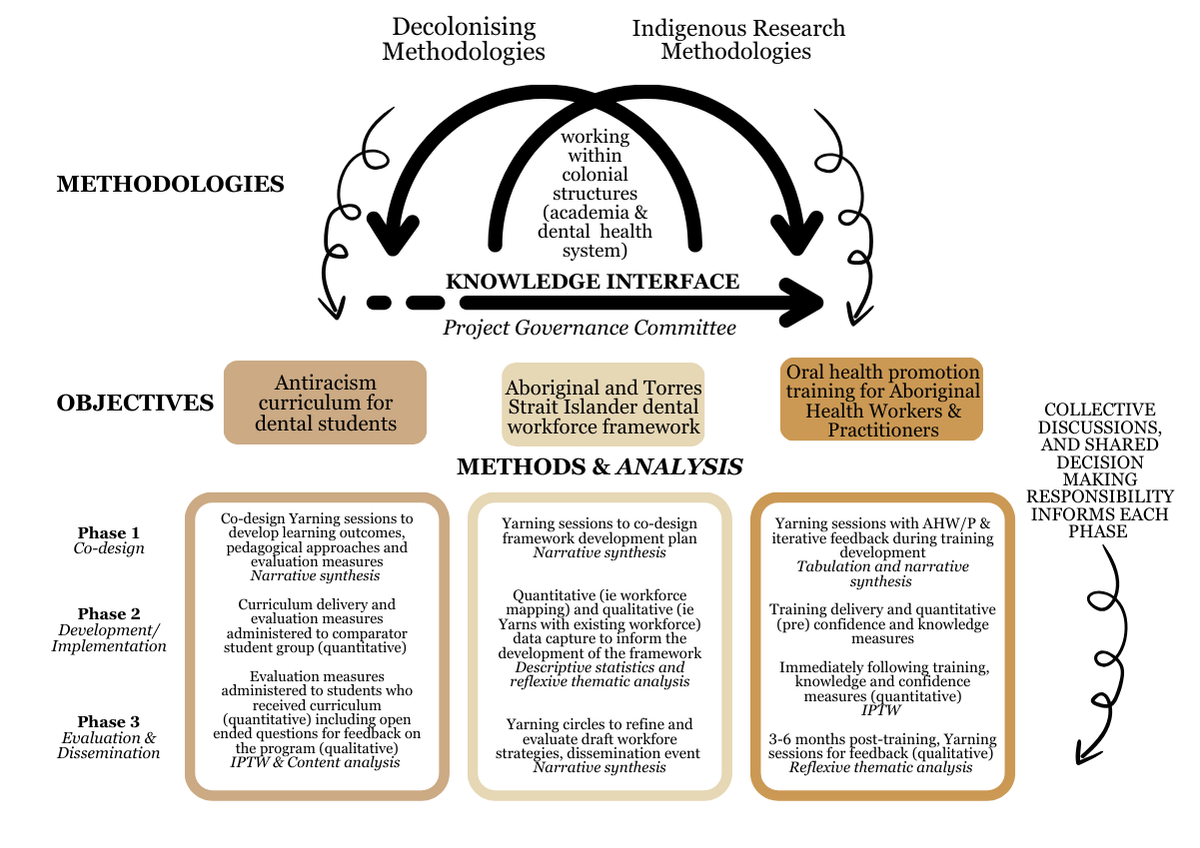
Study design illustrating the interaction between methodologies, methods, and analysis across the objectives and phases of the project. AHW/P: Aboriginal Health Workers/Practitioners; IPTW: inverse probability of treatment weighting.

As a result of relationships and experiences, this project brings together Aboriginal and Torres Strait Islander leadership, lived experiences, Community engagement, and research experience across Australia. The project is grounded in collaborations between the University of Adelaide’s Indigenous Oral health unit, 3 ACCHOs (Yadu Health Aboriginal Corporation in Ceduna, Moorundi Aboriginal Community Controlled Health Service in Murray Bridge, and Umoona Tjutagku Health Service in Coober Pedy), the South Australian Dental Service, the Indigenous Dental Association of Australia (IDAA), the Australian Dental Association, and the Australian Health Practitioner Regulation Agency. The 3 objectives of this study described earlier occur over 3 phases: co-design, implementation, and evaluation and dissemination (Figure 2).

Co-design Yarning sessions will provide the foundation of enquiry for each of our 3 objectives [46]. Multiple cycles of Yarning sessions, as a qualitative research method for data capture, will be required for the iterative co-design process. Yarning sessions will be guided by Aboriginal and Torres Strait Islander team members [48], ensuring the prioritization of Aboriginal and Torres Strait Islander research leadership and engagement, respect, Community benefits, culturally grounded approaches, inclusive partnerships, and robust evidence-based decision-making [53]. In accordance with co-design principles, each Yarning session will be centered around a collective goal, and those engaging in sessions will share the responsibility to prioritize equity, partnership, inclusivity, respect, and trust throughout the sessions [48,53]. Co-design Yarning sessions will be driven by 3 types of evidence: lived experience, professional experience, and academic evidence. Existing evidence pertaining to the collective goal of sessions will be collated by our team and disseminated to co-design participants prior to Yarning sessions. Using the knowledge interface as our guiding methodology, we will bring together lived experiences, Community-driven evidence, academic evidence, and professional experiences to determine appropriate next steps as per the respective objective (Figure 1).

### Objective 1: Development of an Antiracism Curriculum

#### Phase 1: Co-Design Yarning

The co-design Yarning sessions for objective 1 will be an iterative process that actively engages various stakeholders to design and co-construct the curriculum. The collective goal of Yarning sessions will be to develop an antiracism curriculum that equips students to identify racism within the dental care system as well as develop knowledge, skills, and attitudes needed to mitigate racism and its associated consequences. The sessions will be informed by a scoping review of existing antiracism training programs for health trainees (ie, medical and nursing students) and a critical review of evaluation measures of antiracism trainings in health contexts. Overall, the co-design process will be guided by the domains of the Aboriginal and Torres Strait Islander Health Curriculum Framework: respect, communication, safety and quality, reflection, and advocacy; these domains comprise key descriptors such as racism, cultural knowledge, history, strengths-based approaches, equity, and human rights [54]. Working at the knowledge interface, academic evidence from the 2 reviews will be woven together with professional experiences and lived experiences to identify the relevant, theoretically sound, and feasible learning outcomes, pedagogical approaches, and evaluation measures to be included in the curriculum structure.

#### Phase 2: Implementation

Students enrolled in year 4 of the bachelor of dental surgery at the Adelaide Dental School (approximately 70 students) will receive the antiracism curriculum as a core component of the programs. We will pilot the curriculum with students enrolled in 2026, allowing approximately 1 year for curriculum development. The comparator group will be students enrolled in the same year in 2025. The comparator students, all things being equal, will have access to no educational offerings that are discernibly different from the study cohort, apart from receiving the antiracist curriculum. Multiple pedagogical techniques will be used as determined by co-design sessions; this may include cultural immersion, expert-facilitated small group workshops, and clinical simulation activities. Small group tutorials (flipped classroom, with digital short lectures and video or text resources) and simulation exercises will take place in standard tutorial rooms and workshop areas. The curriculum will be delivered by the research team and invited guests who have a combination of lived experiences, expertise in empirical research on racism in health care settings, and extensive training in population health teaching. Those delivering the curriculum will undergo multiple antiracism training programs and will create space for ongoing collective discussions that allow all team members to continue “dialoguing with the tensions” and ensuring decentering of colonial and racialized ideologies [52].

#### Phase 3: Evaluation and Dissemination

Both student groups (2025 and 2026) will complete a set of evaluation measures related to curriculum competencies at the end of their respective years. The evaluation items will be directly informed by the co-design process and related to antiracist behaviors and knowledges, such as the Harvard Implicit Association Test [55] and the Anti-Racism Behavioral Inventory [56]. For students who receive the antiracism curriculum, evaluation will also include open-ended questions that provide an opportunity for feedback on the curriculum [57]. It is important to note that while we will assess knowledge development among students, we are not interested solely in direct comparisons but also in the suitability and sustainability of including antiracism in the curriculum across all dental programs moving forward.

### Objective 2: Supporting the Attraction, Retention, and Well-Being of the Aboriginal and Torres Strait Islander Dental Workforce

#### Phase 1: Co-Design Yarning

Working in partnership with the IDAA, objective 2 will develop meaningful and effective strategies to recruit, support, graduate, and sustain Aboriginal and Torres Strait Islander dental practitioners. Currently, there is minimal infrastructure supporting the Aboriginal and Torres Strait Islander dental workforce within Australia; therefore, co-design Yarning sessions will aim to (1) identify quantitative metrics to be captured with regard to current workforce that can inform strategies (ie, number of Aboriginal and Torres Strait Islander staff within dental schools); (2) identify qualitative data to be collected to inform workforce strategies (ie, directed yarns with existing dental practitioners); and (3) identify key stakeholders important for the dissemination of workforce strategies at the end of the project, so that engagement and relationship building can co-occur throughout development. Prioritizing relationality is key to our collective approach to research and the iterative processes of co-design in this project [30]; hence, the importance of identifying workforce stakeholders at the beginning of the project to ensure relevance of workforce strategies and uptake of recommended strategies among stakeholders. The feedback from these co-design Yarning sessions will directly inform our “development plan” for the workforce strategies.

#### Phase 2: Development

**Figure 3:**
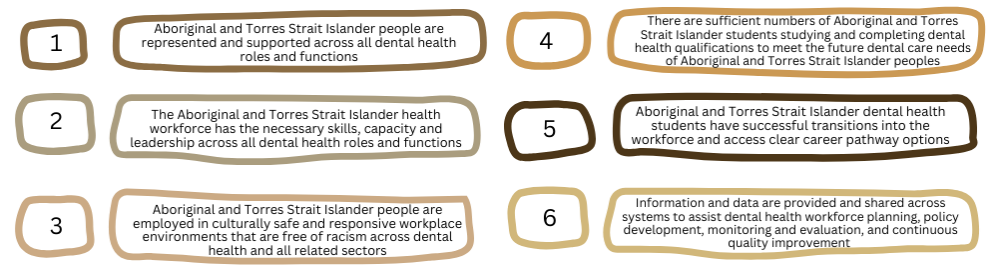
Adapted strategic directions of the National Aboriginal and Torres Strait Islander Health Workforce Strategic Framework and Implementation Plan [27].

Based on collective discussions during co-design Yarning sessions, we will seek to collect data that align with the development plan. We anticipate both qualitative and quantitative data will be collected during the development phase. This will likely include mapping of current Aboriginal and Torres Strait Islander leadership and employment in Australian dental schools and peak dental bodies (quantitative) and Yarning about experiences of Aboriginal and Torres Strait Islander dental practitioners and students with existing support pathways available (qualitative). In an iterative fashion, we will share this data back with IDAA, our PGC, and other stakeholders involved in the initial co-design Yarning sessions. We will organically begin to collate data as related to the 6 strategic directions outlined in the National Aboriginal and Torres Strait Islander Health Workforce Strategic Framework and Implementation Plan [27] (Figure 3). The final step, once all data outlined in the development plan is collected, will be to develop a draft of the workforce strategies, with leadership from IDAA and our PGC.

#### Phase 3: Evaluation and Dissemination

The draft workforce strategies will be shared widely with stakeholders involved throughout development, as well as others with vested interests in the dental workforce. Feedback on the workforce strategies will be garnered in a collective workshop format where everyone comes together to review and share any remaining considerations for the final draft of the workforce strategies. Also at this time, stakeholders will review dissemination plans from the initial co-design Yarning sessions and discuss any other pathways for advocacy and knowledge sharing to support the implementation of the workforce strategies.

### Objective 3: Oral Health Promotion Training

#### Phase 1: Co-Design Yarning

Oral health promotion training for AHW/P is a previously identified Community need [58,59]. We will conduct a scoping review to identify existing oral health promotion materials and previous training programs for Indigenous Health Workers globally. This academic evidence will provide information for co-designing Yarning sessions with AHW/P, whereby both lived experiences and professional experiences will be shared by staff at each of the 3 ACCHO sites. To ensure that this program is relevant and easily integrated by AHW/P, the co-design Yarning sessions will discuss (1) what information is most important to be included in the training; (2) the format of the training sessions; and (3) the evaluation of the training. Support provided for different oral health topics from AHW/P will be tabulated, and qualitative data (audio recordings, text feedback, and field notes) will be narratively synthesized. Data will then be used to develop a training outline, and in alignment with the iterative nature of our co-design approach, we will hold further Yarning sessions with our PGC, ACCHO management, and AHW/P prior to final training development. Information will then be expanded upon as based on the shared outline, and any queries raised during this process will be resolved by the PGC or ACCHO representatives. This iterative process is essential to ensure Aboriginal and Torres Strait Islander leadership drives community-based actions to address oral diseases, in alignment with the National Aboriginal Community Controlled Health Organization (NACCHO) strategic directions [60].

#### Phase 2: Implementation

The pilot of the AHW/P oral health promotion training program will be implemented at 3 partnering ACCHOs in South Australia who have identified the lack of oral health promotion training for their AHW/P staff as a priority area (Yadu Health Aboriginal Corporation in Ceduna, Moorundi Aboriginal Community Controlled Health Service in Murray Bridge, and Umoona Tjutagku Health Service in Coober Pedy). Training will be delivered by team members who have established relationships with AHW/P at each site.

#### Phase 3: Evaluation and Dissemination

Prior to the delivery of the training program, AHW/P knowledge and confidence on promoting oral health will be discussed and recorded via a short questionnaire. Immediately following the delivery of the training program, the same questionnaire will be provided to AHW/P to understand the impacts of the training program. In total, 3-6 months following delivery of the training, Yarning sessions with AHW/P at all sites will be conducted to debrief on the impact of training and specifically understand any enablers and challenges of transferring knowledge gained during the training program into everyday practice. The findings of the training evaluation will be disseminated to peak bodies, including the Aboriginal Health Council of South Australia, the Victorian Aboriginal Community Controlled Health Organization, and NACCHO, to strengthen capacity for advocacy of state-wide and nation-wide implementation of oral health promotion training for AHW/P.

### Ethical Considerations

Ethical approval for this project has been obtained from the Aboriginal Health Research Ethics Committee (#04-23-1085), and the University of Adelaide’s Human Research Ethics Committee (#38988). All study participants will be required to provide written informed consent. Data resulting from the project will be de-identified to protect participant privacy and confidentially. Reimbursement for time will be provided to participants who provide their expertise during co-design Yarning sessions to recognize their valuable contributions (Aus $50 [US $31.02] gift voucher suitable for each region will be provided per engagement in sessions).

### Recruitment

This research will be conducted in partnership with Aboriginal and Torres Strait Islander Peoples, organizations, and stakeholders who have experience and knowledge related to each objective of the project. This will include ACCHO staff, research institutes, universities, training organizations, and national regulatory bodies. Participants for all objectives will be recruited using relational processes across our existing partnerships and networks [48,61]. We will seek to exhaust all relational mechanisms for recruitment and engagement, in alignment with decolonizing methodologies. Participants will be identified and mapped in our stakeholder database with information relating to their cultural and professional backgrounds, including lived experience to clinical and research perspectives, as well as geographic location. As members of the team are introduced to more people working in this space, we will collectively add stakeholders to our database to invite to participate in relevant aspects of the project. Participants for co-design and implementation of objective 3 will be primarily recruited directly through the ACCHO sites (Table 1). Participants who have English as a second or third language will also be invited; interpretation services will be offered to those who want to participate. Participants eligible for participation will be provided with information on participating in this project via email or hard copy depending on context. These documents will outline the purpose of the study, the rights of participants, and details regarding how data will be collected, analyzed, and disseminated.

**Table 1:** Participant eligibility criteria for each project objective.

	Sample size	Participant eligibility criteria
Objective 1	n=50-60 (based on network mapping)	Be aged 18 years and older. Co-design phase: Have expertise and knowledge related to antiracism training or racism within Australia’s health care system, including lived experience, clinical experience, and research experience in this area.
	n=140 (based on 2× typical enrollment)	Implementation phase: Be enrolled in the bachelor of dental science program receiving the pilot antiracist curriculum.
Objective 2	n=70-80 (based on IDAA^a^ data)	Be aged 18 years and older. Identify as an Aboriginal and/or Torres Strait Islander dental stakeholder or occupy a position related to the Aboriginal and/or Torres Strait Islander dental workforce (ie, University dental school dean).
Objective 3	n=40-50 (based on ACCHO^b^ staff)	Be aged 18 years and older. Identify as an Aboriginal and/or Torres Strait Islander AHW/P or hold a management position at an ACCHO.

^a^IDAA: Indigenous Dental Association of Australia.

^b^ACCHO: Aboriginal Community Controlled Health Organization.

### Data Analysis Plan

#### Objective 1

Data from a scoping review of previous antiracism training programs and a critical review of evaluation measures will inform a draft curriculum outline. During the co-design phase, this outline will be reviewed and discussed among key stakeholders for alignment with our 3 types of evidence: lived experiences, professional experiences, and academic evidence. Co-design will be an iterative process whereby feedback is included in the outline following each session for consideration in the following session. Feedback may include consensus from individual sessions to add topics, remove topics, or further explore new or related topics; this feedback will be visually and narratively incorporated into subsequent versions of the outline. Co-design data will either be audio recorded or documented via note-taking during sessions. Once the curriculum outline is established through collective decision-making among those engaged in this process and our PGC, we will revisit co-design Yarning sessions to expand the outline to a full curriculum. This will include discussions about pedagogical approaches and evaluation measures that align with the outline. All data collected throughout this iterative process will then be collated and narratively synthesized [62] to document the co-design process of the curriculum development.

Data captured during the implementation and evaluation phase will be quantitative (both student groups) and qualitative (only the 2026 group that receives training). Both groups will be administered the evaluation measures at the end of the semester of their respective year levels. We anticipate there will be approximately 70 students in each group. Student scores from both years will be adjusted using inverse probability of treatment weighting to account for potential differences in classroom composition, such as average student age, proportion of international students, and gender distribution [63]. Any textual data provided by students who receive the antiracism curriculum will be analyzed using content analysis [64].

#### Objective 2

Data gathered during the co-design phase will be narratively synthesized [62] and will inform the development plan for a framework of workforce strategies. Quantitative data related to mapping of Aboriginal and Torres Strait Islander leadership and employment in the dental health system will be analyzed with descriptive statistics. Directed yarns with Aboriginal and Torres Strait Islander dental practitioners [46] and students will be collectively analyzed by both an Aboriginal and/or Torres Strait Islander researcher and a non-Indigenous researcher using reflexive thematic analysis. Processes of analysis will include data familiarity, extensive discussion with the PGC regarding understandings and assumptions developed during the familiarity process, as well as initial identification of themes during inductive and non-structured coding [65]. These data will then be compared to the 6 strategic directions of the National Aboriginal and Torres Strait Islander Health Workforce Strategic Framework and Implementation Plan [27], and recommendations will initially be co-developed with IDAA and our PGC. Through an iterative process of co-design, stakeholders will be invited to provide feedback on the strategies prior to and during a collective workshop.

#### Objective 3

Data garnered through the co-design phase, including audio recordings, text feedback, and field notes, will be tabulated and narratively synthesized into a training outline. The development will be continuously informed and adjusted through iterative cycles of collective discussions as questions arise during development.

Prior to training delivery, AHW/P knowledge and confidence will be discussed and recorded via a short questionnaire. This same questionnaire will be completed immediately following the training program. Data will be analyzed using inverse probability of treatment weighting, similar to objective 1 [63], to understand any immediate changes among AHW/P as a result of the training. In total, 3-6 months following delivery of the training, yarns with AHW/P [46] will be conducted and audio recorded to understand the usability of the training program in everyday circumstances. Qualitative data from these directed yarns [46] will be analyzed using reflexive thematic analysis, as described earlier [65]. The analysis of the yarns will aim to identify patterns across the data that provide insights into improvements that can strengthen the usefulness of training for AHW/P.

## Results

The study received ethical review approval in February 2024 and received funding in June 2024. The co-design phase for each objective will run from July 2024 to February 2025. The dental curriculum will be developed in 2025 and delivered to the 2026 student cohort. Evaluation data will be collected from the comparator student cohort in 2025 and the implementation cohort in 2026. Data collection for the development of workforce strategies will be collected from October 2024 to July 2025; the framework will be developed from August to December 2025 and disseminated in 2026. Oral health promotion training will be developed from August to February 2024, implemented from March to June 2025, and qualitative evaluation data will be collected between July and September 2025.

## Discussion

### Anticipated Findings

If dental inequities are to be eliminated, comprehensive understanding of the complex factors leading to inequitable oral health outcomes needs to be established. The structural racism embedded in Australia’s dental health system plays a key role in these inequities. The proposed initiative is a novel and innovative project that builds upon over a decade of relationships, collaborations, and engagement with Aboriginal and Torres Strait Islander Community organizations and leaders. The outcomes will directly contribute to fostering an antiracist dental health system that prioritizes cultural safety, quality dental care, and Aboriginal and Torres Strait Islander leadership.

This proposed research will generate evidence and outcomes amenable to translation into policy and practice change, including an antiracist dental curriculum, Aboriginal and Torres Strait Islander dental workforce strategies, and an oral health promotion training for AHW/P. These outcomes will directly enhance the training of non-Indigenous dental health practitioners and support the attraction, retention, and well-being of the Aboriginal and Torres Strait Islander dental workforce and the embedding of oral health within ACCHOs. These strategies will contribute to oral health knowledge at the Community level, in turn supporting Aboriginal and Torres Strait Islander self-determination of oral health. Translation of strategies and learnings will be achieved through dissemination of the research findings to health organizations, services, peak bodies, and universities involved in the research, and subsequent collective advocacy for adoption of evidence into practice guidelines at a practitioner, university, ACCHO, and regulatory level. The collective responsibility and relational nature of this work enhance the feasibility of this work to have tangible impacts across the dental health system in Australia.

### Conclusions

This protocol outlines an antiracist health services and system research project that seeks to intervene at 3 levels of Australia’s dental health system to address structural racism. The findings and materials generated will directly inform policy regarding the development of an antiracist dental health workforce, including antiracist dental practitioners, supportive environments that increase the number of Aboriginal and Torres Strait Islander dental practitioners, and AHW/P equipped with oral health knowledge. The approach used in this project is vital as dental health systems research has predominantly occurred using biomedical, colonial, deficit, and Western perspectives of oral health and healing [66], therefore missing opportunities to address structural racism and resolve oral health inequities effectively, particularly as they pertain to Aboriginal and Torres Strait Islander Peoples [38].

## Appendix

Multimedia Appendix 1 Project governance committee terms of reference.

## Abbreviations

ACCHO: Aboriginal Community Controlled Health Organization
AHW/P: Aboriginal Health Workers/Practitioners
IDAA: Indigenous Dental Association of Australia
NACCHO: National Aboriginal Community Controlled Health Organization
PGC: Project Governance Committee
