# Supplementary material for: Co-Designing an Antiracist Dental Health System: Protocol for an Aboriginal and Torres Strait Islander–Led Mixed Methods Study
Source: JMIR Res Protoc. 2025 May 15;14:e69012. doi: 10.2196/69012 (PMC12123246; doi:10.2196/69012)
Supplement: Multimedia Appendix 1 [file resprot_v14i1e69012_app1.pdf]

Project Governance Committee
Terms of Reference

"The mouth as an expression of racial injustice: Building the evidence to foster an anti-racist dental health system in Australia" (MRF2025028)

PURPOSE

The purpose of the Project Governance Committee (PGC) is to oversee all aspects of the MRFF-funded anti-racism in dentistry project to ensure the project is conducted in a culturally secure manner that prioritises self-determination, demonstrates respect, and privileges Aboriginal and Torres Strait Islander ways of knowing, being and doing and leadership.

MEMBERSHIP

The membership of the Project Governance Committee (PGC) will include representatives from Aboriginal Community Controlled Health Organisations, Aboriginal Health Workers/Practitioners, Elders, Aboriginal and Torres Strait Islander dentists, Aboriginal and Torres Strait Islander dental students, Community leaders, representatives from regulatory and organisational dental bodies, and members of the research team. Aboriginal and Torres Strait Islander members of the PGC will be honoured not only in their roles as academics, health practitioners, or Community leaders but also for their roles as Mothers, Fathers, Aunties, Uncles, and Grandparents with lived experiences and vested interest in the oral health of their families and Communities.

AUTHORITY

The Governance Committee is authorised by the project team to:

1. Review the project's governance matters and make recommendations to the research team

2. Take any action to enable the PGC to fulfil its responsibilities

MEMBERS

Member	Organisation
Director* (Co-chair)	Indigenous Oral Health Unit (IOHU), University of Adelaide

Manager* (Co-chair)	Strategy Partnerships, Women's and Children's Health Network (WCHN)
Professor of Race Relations*	Deakin University
Associate Professor*	Flinders University
Indigenous Data Sovereignty and Governance*	National Indigenous Kidney Transplantation Taskforce, AKction, University of Adelaide
Principal Aboriginal Cultural Learning Consultant*	Women's and Children's Network (WCHN)
Co-president and Director*	Indigenous Dental Association of Australia (IDAA)
Aboriginal Project Officer*	Aboriginal Oral Health Program, SA Dental
National Director*	AHPRA's Aboriginal and Torres Strait Islander Health Strategy Unit
Elder*	Community engagement and Aboriginal and Torres Strait Islander Social and Emotional Wellbeing, Northern Territory
SA Public Sector Anti-Racism Strategy project team*	Preventive Health SA
Indigenous Research Officers*	Indigenous Oral Health Unit (IOHU), University of Adelaide
Aboriginal Project Officer*	Aboriginal Oral Health Program, South Australian Dental Service
Indigenous Dental Practitioners*	University of Adelaide
Senior Lecturer – Indigenous Health*	Indigenous Health, Adelaide Medical School
Clinic director*	Moorundi Aboriginal Community Controlled Health Service Inc
Clinic manager*	Moorundi Aboriginal Community Controlled Health Service Inc
Clinic manager*	Yadu Health
CEO*	Umoona Tjutagku Health Service
Project leads	Indigenous Oral Health Unit (IOHU), University of Adelaide
Director	ARCPOH, University of Adelaide

**Aboriginal and/or Torres Strait Islander*

EXECUTIVE DECISION MAKING GROUP

In circumstances whereby a decision needs to be made quickly or immediately (for example, a decision to travel (or not) to a Community during Sorry Business), a small Executive Decision Making Group, consisting of 3-4 people will advise the research team on the best way forward. This Executive Decision Making Group will consist of the PGC co-chairs and 2 individuals elected from the floor, rotating on an annual basis.

MEETINGS

Meetings will be held quarterly each year, for the duration of the project at times determined by the committee members. The inaugural meeting will take place on Kaurna Country on June 25, 2024.

Any aspects of the project that need to be reviewed outside of PGC meetings will be sent via email with clear instructions on feedback being sought and any associated deadlines. Offline review will be based on individual availability and expertise on topic.

RESPONSIBILITIES

The PGC is responsible for advising the research team on project processes, milestones, and outputs through:

- Reviewing yarning guides, publications, visuals and presentations
- Participant recruitment
- Involvement in co-design processes
- Feedback on student work
- Provide offline expertise-based guidance as needed
- Share knowledge about the project with networks and Communities

ACCOUNTABILITY

The PGC is accountable to the project team for the following tasks:

- Quarterly assessment of the project's performance, including strengths, weaknesses and skills of the team as a whole
- Recommending a plan for project development based on milestones and expected outcomes
- Ongoing recruitment of team members and participants that fulfil project needs
- Mentoring new team members in order to develop their skillset
- Drafting governance procedures
- Minutes of PGC meetings will be circulated to all members of the Committee and research team

ABOUT THE PROJECT

This project ("The mouth as an expression of racial injustice: Building the evidence to foster an anti-racist dental health system in Australia") has been funded by the 2022 Medical Research Future Fund Indigenous Health Research Grant (MRF2025028) and is being led by the Indigenous Oral Health Unit at the University of Adelaide. Ethical approval has been obtained from the Aboriginal Human Research Ethics Committee (#04-23-1085) and the University of Adelaide Human Research Ethics.

The project aims to build evidence to foster an anti-racist dental health system in Australia, through the following objectives:

1. Co-design and implement an **anti-racist curriculum** for dental students that can be robustly evaluated.

2. We will **support the attraction, retention, and wellbeing** of the Aboriginal and Torres Strait Islander dental workforce.

3. Develop an **oral health promotion training** module for Aboriginal Health Workers and Practitioners, that can be comprehensively assessed and disseminated to Aboriginal Community Controlled Health Services.
